# Supplementary figures and images for: The chromatin-remodeling enzyme Smarca5 regulates erythrocyte aggregation via Keap1-Nrf2 signaling
Source: eLife. 2021 Oct 26;10:e72557. doi: 10.7554/eLife.72557 (PMC8594921; doi:10.7554/eLife.72557)

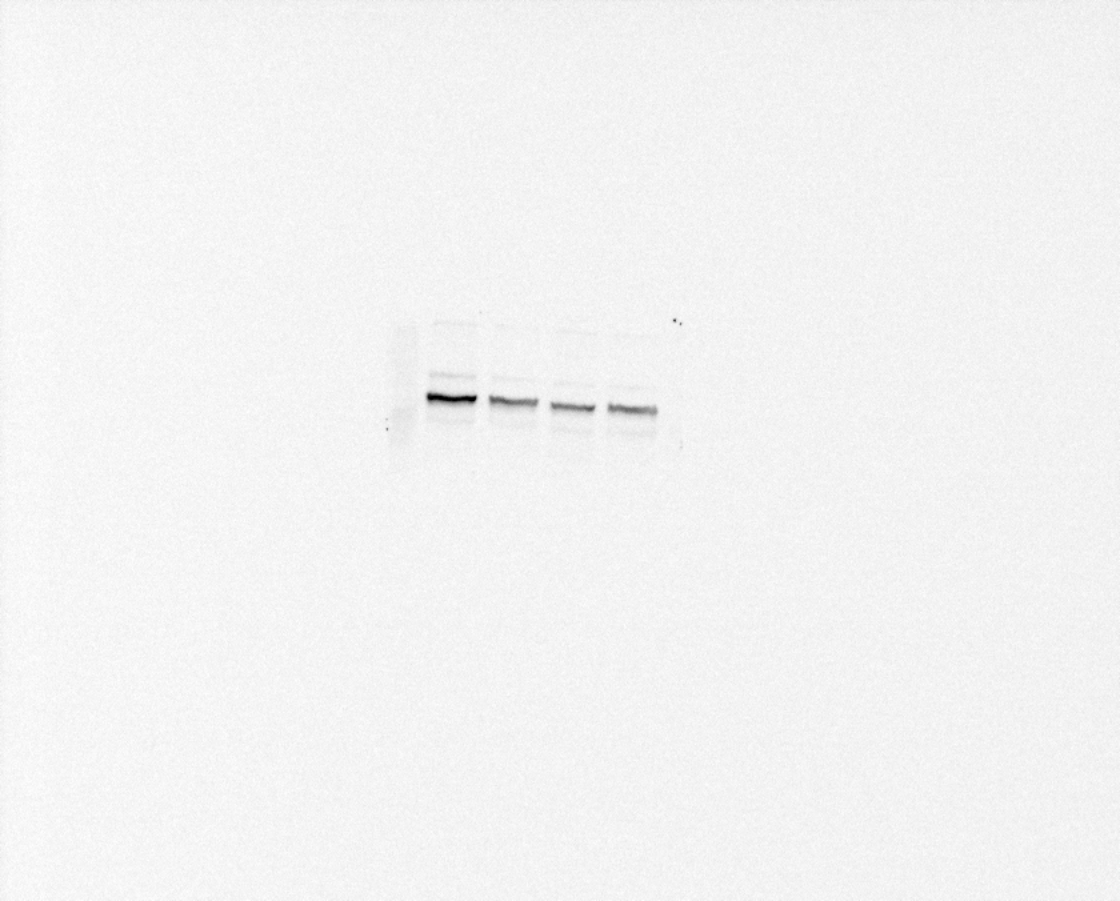

Supplement: Figure 6—figure supplement 1—source data 1. [file elife-72557-fig6-figsupp1-data1.zip › Figure6S1SourceData1a.tif]

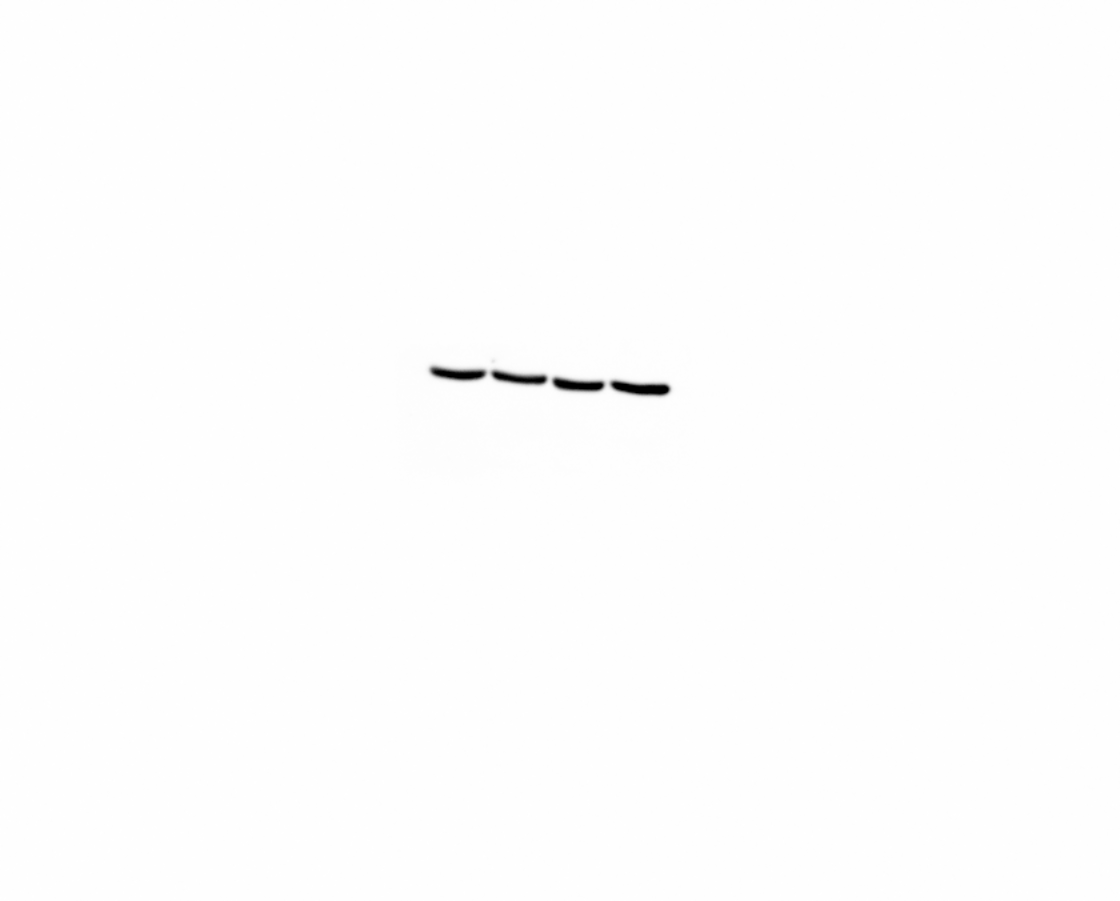

Supplement: Figure 6—figure supplement 1—source data 1. [file elife-72557-fig6-figsupp1-data1.zip › Figure6S1SourceData1b.tif]

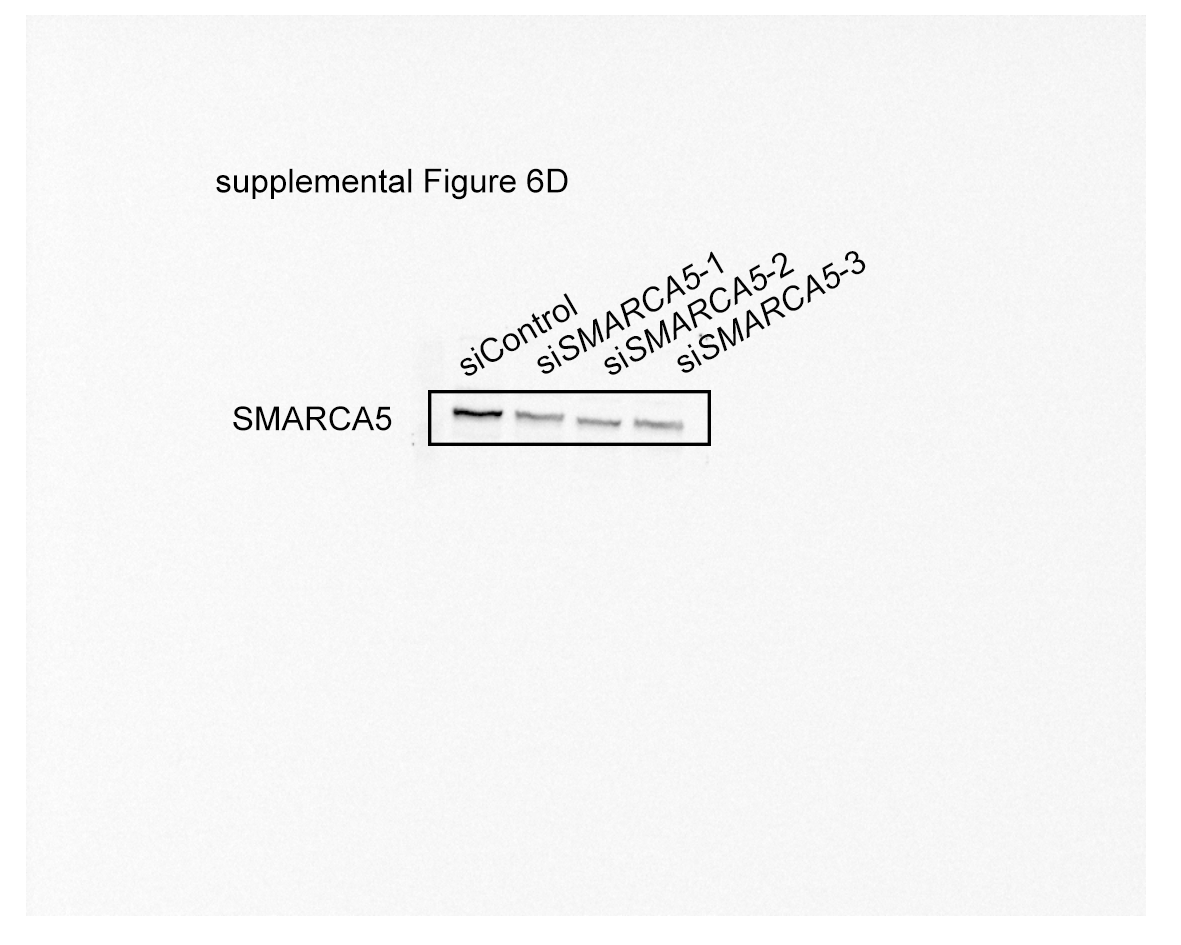

Supplement: Figure 6—figure supplement 1—source data 2. [file elife-72557-fig6-figsupp1-data2.zip › Figure6S1SourceData2a.tif]

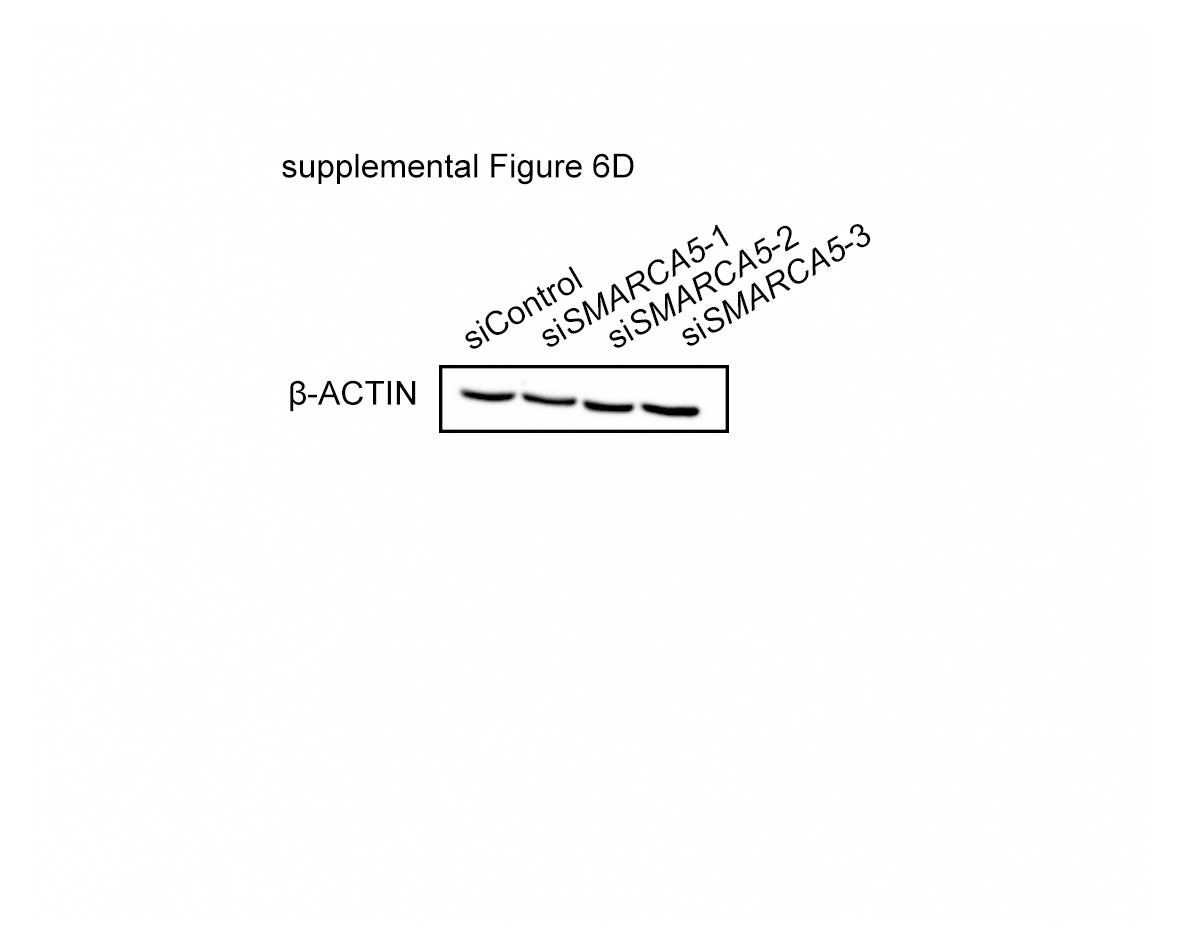

Supplement: Figure 6—figure supplement 1—source data 2. [file elife-72557-fig6-figsupp1-data2.zip › Figure6S1SourceData2b.tif]
