# Supplementary material for: The chromatin-remodeling enzyme Smarca5 regulates erythrocyte aggregation via Keap1-Nrf2 signaling
Source: eLife. 2021 Oct 26;10:e72557. doi: 10.7554/eLife.72557 (PMC8594921; doi:10.7554/eLife.72557)
Supplement: Supplementary file 1. — (A) The detailed primers used for qPCR. (B) The detailed sequence and dosage of MOs used in this work. (C) The detailed sequences of siRNAs used in this work. [file elife-72557-supp1.docx]

**Supplementary file 1A. The PCR primers used for qPCR.**

| Primer name | Sequence (5'-3') | Reference |
| --- | --- | --- |
| *β-actin*-qPCR-F | GCTGTTTTCCCCTCCATTGTT | (Zhang et al., 2017) |
| *β-actin*-qPCR-R | TCCCATGCCAACCATCACT |  |
| *gata1*-qPCR-F | TCTGAGCCTTCTCGTTGGG | (Ren et al., 2013) |
| *gata1*-qPCR-R | CTCTGGACGCTGGTGGAATA |  |
| *ikaros*-qPCR-F | TAACCTGCTCCGACACAT | (Wang, He, Ma, Xue, & Liu, 2015) |
| *ikaros*-qPCR-R | CTCCGCTGCTTGTAACTG |  |
| *hbae1*-qPCR-F | GCAAAGTCATCCTTCCAC | (Xue, Gao, & Liu, 2015) |
| *hbae1*-qPCR-R | GAAGTAGGTCTTGGTCTGG |  |
| *hbbe1*-qPCR-F | CTGAACATCTAAAGTCGAAAA | (Xue et al., 2015) |
| *hbbe1*-qPCR-R | ACACCTTGCCAGAGCCT |  |
| *pu.1*-qPCR-F | AGGAGTGTATGAGAGACCACATCAG | (Ren et al., 2013) |
| *pu.1*-qPCR-R | ATTTCGCAGAAGGTCAAGCA |  |
| *mfap4*-qPCR-F | ATGGACGGCAGTGTGAAT | (Wang et al., 2015) |
| *mfap4*-qPCR-R | CTTCTTGTGGCGTGTCAG |  |
| *lyz*-qPCR-F | GTGAAAATGGACGGGCTGAA | (Wang et al., 2015) |
| *lyz*-qPCR-R | CTTTGTTTGCGCTGCTCACA |  |
| *keap1a*-qPCR-F | GCACTGACCTACACCTTCGC | (Liu et al., 2019) |
| *keap1a*-qPCR-R | GCCTTGTAGACCTCGCTCTC |  |
| *hmox1a*-qPCR-F | GCTTCTGCTGTGCTCTCTATACG | (Holowiecki, O'Shields, & Jenny, 2017) |
| *hmox1a*-qPCR-R | CAATCTCTCTCAGTCTCTGTGC |  |
| *gclc*-qPCR-F | AACCGACACCCAAGATTCAGCACT | (Timme-Laragy, Van Tiem, Linney, & Di Giulio, 2009) |
| *gclc*-qPCR-R | CCATCATCCTCTGGAAACACCTCC |  |
| *ggt1b*-qPCR-F | ATCAATGCTCGTGAGACAGC | (Sant et al., 2017) |
| *ggt1b*-qPCR-R | TGGACAAACCACCTTTCTGG |  |
| *gsr*-qPCR-F | ATGTGCCAGGATCCAGTTTAG | (Sant et al., 2017) |
| *gsr*-qPCR-R | ACCCAAGAGTGGAAATACC |  |
| *gstp1*-qPCR-F | CGACTTGAAAGCCACCTGTGTC | (Timme-Laragy et al., 2012) |
| *gstp1*-qPCR-R | CTGTCGTTTTTGCCATATGCAGC |  |
| *gstk1*-qPCR-F | TACTTTGGGGTTCCTGTGCG | (Liu et al., 2019) |
| *gstk1*-qPCR-R | TCTCCTTCTCTGCTACCGCT |  |
| *gsto2*-qPCR-F | ATGGCTTCATCTCCAAAATGC | (Nguyen et al., 2016) |
| *gsto2*-qPCR-R | AGGGCAGAATCTCATGCTGTAG |  |
| *prdx1*-qPCR-F | GCCCGCGAGTTCACTTTC | (Nakajima et al., 2011) |
| *prdx1*-qPCR-R | GCTTCCATCCGGCTGGAC |  |
| *g6pd*-qPCR-F | ATGATGTGCGGGATGAGAAGGTAAA | (Holowiecki et al., 2017) |
| *g6pd*-qPCR-R | TGCTGTGGCAAATGTAGCCTGAGT |  |
| *pgd*-qPCR-F | TGGAATACGGCACACCTGTC | (Nguyen et al., 2016) |
| *pgd*-qPCR-R | AGGCTCTTACTGGCCTGAAC |  |
| *fbp1a*-qPCR-F | ATCGTTGTAGAGCCAGATAGAC | (Nguyen et al., 2016) |
| *fbp1a*-qPCR-R | TGTAGATGGCAAAGATGGTCC |  |
| human*SMARCA5*-qPCR-F | AACTTACTATCCGTTGGCGATT | (Zikmund et al., 2020) |
| human*SMARCA5*-qPCR-R | GGTTGCTTTGGAGCTTTCTG |  |
| human*HMOX1*-qPCR-F | TTCAAGCAGCTCTACCGCTC | (Chen et al., 2019) |
| human*HMOX1*-qPCR-R | GAACGCAGTCTTGGCCTCTT |  |
| human*KEAP1*-qPCR-F | AACGGTGCTGTCATGTACCA | (Tian et al., 2020) |
| human*KEAP1*-qPCR-R | GGCAGTGGGACAGGTTGAA |  |
| human*NRF2*-qPCR-F | TCCAGTCAGAAACCAGTGGAT | (Tian et al., 2020) |
| human*NRF2*-qPCR-R | GAATGTCTGCGCCAAAAGCTG |  |
| human*GADPH*-qPCR-F | AGCCACATCGCTCAGACAC | (Zikmund et al., 2020) |
| human*GADPH*-qPCR-R | GCCCAATACGACCAAATCC |  |

**Supplementary file 1B. The MOs used in this work.**

| Gene name | Sequence (5'-3') | Dosage (ng/embryo) | Reference |
| --- | --- | --- | --- |
| *pu.1* MO | CCTCCATTCTGTACGGATGCAGCAT | 4 | (Clay et al., 2007) |
| *hmox1a* MO | AGTCCATCTTTGTGCTGTAGATGTC | 1 | (Tzaneva & Perry, 2016) |

**Supplementary file 1C. The siRNAs used in this work.**

| siRNA | Sequence (5'-3') | Reference |
| --- | --- | --- |
| siControl | UUCUCCGAACGUGUCACGUTT | This work |
| si*SMARCA5*-1 | CCGGGCAAAUAGAUUCGAGUAUUUA | This work |
| si*SMARCA5*-2 | AAGAGGAGGAUGAAGAGCUAU | (Collins et al., 2002) |
| si*SMARCA5*-3 | GUUCUUUCCUCCACGUUUA | (Bryant, Colgrove, & Knipe, 2011) |

**Supplemental references**

Bryant, K. F., Colgrove, R. C., & Knipe, D. M. (2011). Cellular SNF2H chromatin-remodeling factor promotes herpes simplex virus 1 immediate-early gene expression and replication. *mBio, 2*(1), e00330-00310. doi:10.1128/mBio.00330-10

Chen, Z., Zhong, H., Wei, J., Lin, S., Zong, Z., Gong, F., Huang, X., Sun, J., Li, P., Lin, H., Wei, B., & Chu, J. (2019). Inhibition of Nrf2/HO-1 signaling leads to increased activation of the NLRP3 inflammasome in osteoarthritis. *Arthritis Res Ther, 21*(1), 300. doi:10.1186/s13075-019-2085-6

Clay, H., Davis, J. M., Beery, D., Huttenlocher, A., Lyons, S. E., & Ramakrishnan, L. (2007). Dichotomous role of the macrophage in early Mycobacterium marinum infection of the zebrafish. *Cell Host Microbe, 2*(1), 29-39. doi:10.1016/j.chom.2007.06.004

Collins, N., Poot, R. A., Kukimoto, I., Garcia-Jimenez, C., Dellaire, G., & Varga-Weisz, P. D. (2002). An ACF1-ISWI chromatin-remodeling complex is required for DNA replication through heterochromatin. *Nat Genet, 32*(4), 627-632. doi:10.1038/ng1046

Holowiecki, A., O'Shields, B., & Jenny, M. J. (2017). Spatiotemporal expression and transcriptional regulation of heme oxygenase and biliverdin reductase genes in zebrafish (Danio rerio) suggest novel roles during early developmental periods of heightened oxidative stress. *Comp Biochem Physiol C Toxicol Pharmacol, 191*, 138-151. doi:10.1016/j.cbpc.2016.10.006

Liu, Y., Liu, X., Wang, Y., Yi, C., Tian, J., Liu, K., & Chu, J. (2019). Protective effect of lactobacillus plantarum on alcoholic liver injury and regulating of keap-Nrf2-ARE signaling pathway in zebrafish larvae. *Plos One, 14*(9), e0222339. doi:10.1371/journal.pone.0222339

Nakajima, H., Nakajima-Takagi, Y., Tsujita, T., Akiyama, S., Wakasa, T., Mukaigasa, K., Kaneko, H., Tamaru, Y., Yamamoto, M., & Kobayashi, M. (2011). Tissue-restricted expression of Nrf2 and its target genes in zebrafish with gene-specific variations in the induction profiles. *Plos One, 6*(10), e26884. doi:10.1371/journal.pone.0026884

Nguyen, V. T., Fuse, Y., Tamaoki, J., Akiyama, S. I., Muratani, M., Tamaru, Y., & Kobayashi, M. (2016). Conservation of the Nrf2-Mediated Gene Regulation of Proteasome Subunits and Glucose Metabolism in Zebrafish. *Oxid Med Cell Longev, 2016*, 5720574. doi:10.1155/2016/5720574

Ren, C. G., Wang, L., Jia, X. E., Liu, Y. J., Dong, Z. W., Jin, Y., Chen, Y., Deng, M., Zhou, Y., Zhou, Y., Ren, R. B., Pan, W. J., & Liu, T. X. (2013). Activated N-Ras signaling regulates arterial-venous specification in zebrafish. *J Hematol Oncol, 6*, 34. doi:10.1186/1756-8722-6-34

Sant, K. E., Hansen, J. M., Williams, L. M., Tran, N. L., Goldstone, J. V., Stegeman, J. J., Hahn, M. E., & Timme-Laragy, A. (2017). The role of Nrf1 and Nrf2 in the regulation of glutathione and redox dynamics in the developing zebrafish embryo. *Redox Biol, 13*, 207-218. doi:10.1016/j.redox.2017.05.023

Tian, Y., Liu, Q., Yu, S., Chu, Q., Chen, Y., Wu, K., & Wang, L. (2020). NRF2-Driven KEAP1 Transcription in Human Lung Cancer. *Mol Cancer Res, 18*(10), 1465-1476. doi:10.1158/1541-7786.MCR-20-0108

Timme-Laragy, A. R., Karchner, S. I., Franks, D. G., Jenny, M. J., Harbeitner, R. C., Goldstone, J. V., McArthur, A. G., & Hahn, M. E. (2012). Nrf2b, novel zebrafish paralog of oxidant-responsive transcription factor NF-E2-related factor 2 (NRF2). *J Biol Chem, 287*(7), 4609-4627. doi:10.1074/jbc.M111.260125

Timme-Laragy, A. R., Van Tiem, L. A., Linney, E. A., & Di Giulio, R. T. (2009). Antioxidant responses and NRF2 in synergistic developmental toxicity of PAHs in zebrafish. *Toxicol Sci, 109*(2), 217-227. doi:10.1093/toxsci/kfp038

Tzaneva, V., & Perry, S. F. (2016). Evidence for a role of heme oxygenase-1 in the control of cardiac function in zebrafish (Danio rerio) larvae exposed to hypoxia. *J Exp Biol, 219*(Pt 10), 1563-1571. doi:10.1242/jeb.136853

Wang, S., He, Q., Ma, D., Xue, Y., & Liu, F. (2015). Irf4 Regulates the Choice between T Lymphoid-Primed Progenitor and Myeloid Lineage Fates during Embryogenesis. *Developmental Cell, 34*(6), 621-631. doi:10.1016/j.devcel.2015.07.011

Xue, Y., Gao, S., & Liu, F. (2015). Genome-wide analysis of the zebrafish Klf family identifies two genes important for erythroid maturation. *Dev Biol, 403*(2), 115-127. doi:10.1016/j.ydbio.2015.05.015

Zhang, C., Chen, Y., Sun, B., Wang, L., Yang, Y., Ma, D., Lv, J., Heng, J., Ding, Y., Xue, Y., Lu, X., Xiao, W., Yang, Y. G., & Liu, F. (2017). m(6)A modulates haematopoietic stem and progenitor cell specification. *Nature, 549*(7671), 273-276. doi:10.1038/nature23883

Zikmund, T., Paszekova, H., Kokavec, J., Kerbs, P., Thakur, S., Turkova, T., Tauchmanova, P., Greif, P. A., & Stopka, T. (2020). Loss of ISWI ATPase SMARCA5 (SNF2H) in Acute Myeloid Leukemia Cells Inhibits Proliferation and Chromatid Cohesion. *Int J Mol Sci, 21*(6). doi:10.3390/ijms21062073
